# Genetic polymorphisms that affect selenium status and response to selenium supplementation in United Kingdom pregnant women^1^

**DOI:** 10.3945/ajcn.115.114231

**Published:** 2015-12-16

**Authors:** Jinyuan Mao, Jessica J Vanderlelie, Anthony V Perkins, Christopher WG Redman, Kourosh R Ahmadi, Margaret P Rayman

**Affiliations:** 2 Department of Endocrinology and Metabolism, the First Hospital of China Medical University, Shenyang, China;; 3 Department of Nutritional Sciences, Faculty of Health and Medical Sciences, University of Surrey, Guildford, United Kingdom;; 4 School of Medical Science, Griffith Health Institute, Griffith University, Queensland, Australia; and; 5 Nuffield Department of Obstetrics and Gynecology, University of Oxford, Oxford, United Kingdom

**Keywords:** DMGDH, polymorphisms, pregnancy, selenium status, SEPP1

## Abstract

**Background:** Low selenium status in pregnancy has been associated with a number of adverse conditions. In nonpregnant populations, the selenium status or response to supplementation has been associated with polymorphisms in dimethylglycine dehydrogenase (*DMGDH*), selenoprotein P (*SEPP1*) and the glutathione peroxidases [cytosolic glutathione peroxidase (*GPx1*) and phospholipid glutathione peroxidase (*GPx4*)].

**Objective:** We hypothesized that, in pregnant women, these candidate polymorphisms would be associated with selenium status in early pregnancy, its longitudinal change, and the interindividual response to selenium supplementation at 60 μg/d.

**Design:** With the use of stored samples and data from the United Kingdom Selenium in Pregnancy Intervention (SPRINT) study in 227 pregnant women, we carried out genetic-association studies, testing for associations between selenium status, its longitudinal change, and response to supplementation and common genetic variation in *DMGDH* (rs921943), *SEPP1* (rs3877899 and rs7579), *GPx1* (rs1050450) and *GPx4* (rs713041). Selenium status was represented by the concentration of whole-blood selenium at 12 and 35 wk of gestation, the concentration of toenail selenium at 16 wk of gestation, and plasma glutathione peroxidase (GPx3) activity at 12 and 35 wk of gestation.

**Results:** Our results showed that *DMGDH* rs921943 was significantly associated with the whole-blood selenium concentration at 12 wk of gestation (*P* = 0.032), which explained ≤2.0% of the variance. This association was replicated with the use of toenail selenium (*P* = 0.043). In unsupplemented women, *SEPP1* rs3877899 was significantly associated with the percentage change in whole-blood selenium from 12 to 35 wk of gestation (*P* = 0.005), which explained 8% of the variance. In supplemented women, *SEPP1* rs3877899 was significantly associated with the percentage change in GPx3 activity from 12 to 35 wk of gestation (*P* = 0.01), which explained 5.3% of the variance. Selenium status was not associated with *GPx1*, *GPx4*, or *SEPP1* rs7579.

**Conclusions:** In agreement with previous studies, we show that the genetic variant rs921943 in *DMGDH* is significantly associated with selenium status in United Kingdom pregnant women. Notably, our study shows that women who carry the *SEPP1* rs3877899 A allele are better able to maintain selenium status during pregnancy, and their GPx3 activity increases more with supplementation, which suggests better protection from low selenium status. The SPRINT study was registered at www.isrctn.com as ISRCTN37927591.

## INTRODUCTION

Studies have shown an association or correlation between low selenium status in pregnancy (toenail or circulating selenium concentrations) and pre-eclampsia (1, 2), pregnancy-induced hypertension (2), preterm birth (3), glucose intolerance (4), a more adverse lipid profile (5), and greater inflammation (high-sensitivity C-reactive protein) (5). In contrast, higher selenium status in pregnant women that resulted from selenium supplementation reduced risks of pre-eclampsia (6), pregnancy-induced hypertension (2) and, in women with autoimmune thyroiditis, thyroid inflammatory activity (7). These findings make it clear that selenium status in pregnancy is a variable that deserves to be investigated and understood.

Selenium status is assessed in a number of ways (8, 9) including the plasma or serum selenium concentration (reflecting recent intake), the whole-blood selenium concentration (a longer-term measure reflecting the 120-d lifetime of red blood cells), the toenail selenium concentration [an even longer-term measure that integrates status over 6–12 mo (10) and is backdated to when the nails were laid down], and functional measures of selenium status that assess the concentration or activity of selenoproteins and selenoenzymes (e.g., glutathione peroxidase activity in plasma [plasma glutathione peroxidase (GPx3)^5^]).

The most important determinant of selenium status is intake of selenium through the diet or supplements (11), but other factors have been reported to be associated with status such as sex (12), age (13, 14), menopausal status (14), BMI (12), education (14), smoking (13, 14), social class and socioeconomic status (2, 13, 14), frailty or illness (13), and genotype (12, 15, 16).

Recent genome-wide association studies have revealed a significant link between a locus at chromosome 5q14 and erythrocyte (15) and toenail-selenium (16) concentrations. In the first study, the most significant association was shown with rs921943 in dimethylglycine dehydrogenase (*DMGDH*; coding for DMGDH) (15), whereas in the second study, the most-significant association was with rs17823744 in the same gene (16); there is some linkage disequilibrium between these single nucleotide polymorphisms (SNPs) [*D*′ = 0.803, *r*^2^ = 0.28; from HapMap (data release 28 (NCBI Build 36 Assembly) Caucasian (CEU) data], and a sample-weighted meta-analysis across both studies revealed the most significant locus to be rs921943 (16). DMGDH is a protein involved in the metabolism of sulfur-containing amino acids, including methionine, and, potentially, of the analogous selenium compounds such as selenomethionine (15, 16) which is the most common selenium species in food sources (17).

Four other polymorphisms have also been shown to affect plasma selenium or selenoprotein concentrations or the selenoprotein concentration or activity in response to supplementation (12, 18, 19). These polymorphisms are selenoprotein P (*SEPP1)* rs3877899 and rs7579, cytosolic glutathione peroxidase (*GPx1*) rs1050450, and phospholipid glutathione peroxidase (*GPx4*) rs713041 (see **Table 1** for their characteristics and the functions of the proteins they encode).

**TABLE 1:** Characteristics of candidate SNPs investigated and functions of the proteins they encode^1^

Chromosome	Gene	Function	SNP	Position^2^	SNP location	Allele, major/minor	MAF, fraction	Genotype count	*P*-HWE
5q14	*DMGDH*	Encodes the enzyme DMGDH, which is involved in the catabolism of choline that catalyzes the oxidative demethylation of dimethylglycine to form sarcosine. Linked to 1-C metabolism.	rs921943	79020653	Intron	G/A	0.302	111/95/21	0.876
5q31	*SEPP1*	Encodes the extracellular SEPP1 that contains multiple selenocysteine residues. Transports selenium in the bloodstream to tissues such as the brain, testis, and placenta. Functions as an antioxidant in the extracellular space.	rs3877899	42801166	Ala234Thr	G/A	0.239	131/79/14	0.713
5q31	*SEPP1*		rs7579	42800706	3′UTR	G/A	0.280	121/82/22	0.184
3p21	*GPx1*	Encodes GPx1, which detoxifies hydrogen peroxide and lipid hydroperoxides and is one of the most-important antioxidant enzymes in humans.	rs1050450	49357401	Pro198Leu	C/T	0.324	104/96/25	0.761
19p13	*GPx4*	Encodes GPx4, which can reduce hydrogen peroxide and lipid hydroperoxides in biological membranes.	rs713041	1106616	3′UTR	C/T	0.456	63/119/43	0.350

1 DMGDH, dimethylglycine dehydrogenase; GPx1, cytosolic glutathione peroxidase; GPx4, phospholipid glutathione peroxidase; HWE, Hardy-Weinberg equilibrium; MAF, minor allele frequency; SEPP1, selenoprotein P; SNP, single nucleotide polymorphism; UTR, untranslated region.

2 From the dbSNP database http://www.ncbi.nlm.nih.gov/snp (accessed 21 April 2015).

To our knowledge, no previous study has looked at the effect of these polymorphisms on selenium status during pregnancy. To address this issue, we used stored samples that were suitable for DNA genotyping and previous measurements of a number of variables of selenium status from SPRINT (Selenium in Pregnancy Intervention), which is a United Kingdom study in pregnant women (6). Having relatively low whole-blood and toenail selenium, United Kingdom pregnant women (1, 2, 6) are suitable subjects for such an investigation. As markers of selenium status, we used the whole-blood selenium concentration at 12 and 35 wk of gestation, the toenail selenium concentration at 16 wk of gestation, and the activity of GPx3 at 12 and 35 wk of gestation. Baseline samples were collected at 12 wk of gestation or at 16 wk of gestation for toenail clippings.

We hypothesized that one or more of the candidate SNPs would be significantly associated with *1*) the whole-blood or toenail selenium concentration at baseline, *2*) the longitudinal change in whole-blood selenium over the course of pregnancy, and *3*) the response of whole-blood selenium or GPx3 activity to selenium supplementation.

## METHODS

### Participants

Biological samples for this study originated from the SPRINT study (www.isrctn.com; ISRCTN37927591). The selection of subjects has been described previously (6). Women were excluded if they were <18 y old, current smokers, taking any supplement containing selenium, taking thyroid medication, had a multifetal pregnancy or a number of other specified pregnancy complications, or withheld consent. Primiparous women (*n* = 230) in Oxford, United Kingdom, were randomly assigned to treatment with selenium (60 μg Se/d as selenium yeast) or a placebo (placebo yeast) from their first hospital antenatal visit (mean ± SD gestational age: 12.3 ± 0.9 wk) until delivery of their babies (6). Blood samples, from which plasma was prepared, were collected at baseline (12 wk of gestation) and 35 wk of gestation, whereas toenail clippings were collected at 16 wk of gestation as described previously (6). One woman in the placebo group was recruited in error (was receiving treatment with thyroxine) and was excluded from the analysis as were 2 women in the selenium group (one woman was a selenium-status outlier, and one woman withheld consent for genotyping). For the analysis, there remained 114 women in the placebo group and 113 women in the selenium group at baseline and 109 women in the placebo group and 104 women in the selenium group at 35 wk of gestation (6).

The study was conducted according to the guidelines of the Declaration of Helsinki, and all procedures involving human subjects were approved by the Milton Keynes Research Ethics Committee (reference 08/H0603/46). Written informed consent was obtained from all subjects.

### Laboratory analyses

Whole-blood selenium was determined at 12 and 35 wk of gestation with the use of inductively coupled plasma mass spectrometry at the Trace Element Unit, Southampton University Hospital National Health Service Trust as previously described (6). GPx3 activity at 12 and 35 wk of gestation was determined with the use of a spectrophotometric assay as previously described (2). Toenail selenium concentrations were measured in clippings collected from all 10 toes at 16 wk of gestation by instrumental neutron activation analysis as previously described (1).

### Genotyping

DNA was extracted from baseline whole-blood samples, which were stored at −80°C with the use of a FlexiGene DNA kit (QIAGEN) according to the manufacturer’s instructions. The genotyping of rs921943 in *DMGDH*, rs3877899 and rs7579 in *SEPP1*, rs1050450 in *GPx1*, and rs713041 in *GPx4* was completed with the use of KASP assays at LGC Genomics. The call rate of all SNPs was > 98%, and none deviated from Hardy-Weinberg equilibrium (*P* > 0.05).

### Statistical analysis

All outcome variables (whole-blood selenium, toenail selenium, and GPx3 activity) at baseline were log transformed and tested for normality before the analysis. Log transformation produced distributions that were normal in the case of log whole-blood selenium and log toenail selenium and approximately normal in the case of GPx3; all residuals were normally distributed.

To explore the effect of genotype during pregnancy on longitudinal changes in whole-blood selenium and GPx3 activity in the placebo group and the response to selenium supplementation in the selenium-intervention group, percentage changes in whole-blood selenium and in GPx3 activity from 12 to 35 wk of gestation were calculated. Baseline age, gestational age, BMI, age at which education ceased (y), and social class [dichotomized as previously reported (2)], all of which have been shown to be associated with selenium status (12–14), were used as covariates as appropriate.

All genetic analyses was carried out with the use of the PLINK v1.9 analysis toolset (20, 21), which was specifically designed to perform a range of genetic analyses through a flexible regression framework. The flexibility of the analysis framework allows for covariates (e.g., age and BMI) to be readily included and tested for an association with baseline variability, the longitudinal change, and the response to selenium supplementation. We tested each SNP for Hardy-Weinberg equilibrium with the use of the full cohort. A type I error rate was controlled for with the use of Bonferroni correction with the adjusted statistical threshold of significance: α′ = α ÷ *n*, where α is the nominal significance threshold for a single test (α =0.05), and *n* equals 5 (i.e., the total number of genotyped SNPs tested). We present nominal *P* values and draw attention to significant adjusted values (*P* < 0.01) where appropriate.

## RESULTS

The age at which education ceased (y) and social class (dichotomized) were significantly associated with selenium status at baseline in the univariate analysis but not with percentage changes in whole-blood selenium and GPx3 activity. Therefore, they were adjusted for in the analysis of selenium status at baseline but not in the analysis of the percentage change in whole-blood selenium and GPx3 activity.

### Association between SNPs and selenium status at baseline

Characteristics of candidate SNPs including the position, function, minor allele frequencies, genotype counts, and *P* value for Hardy-Weinberg equilibrium are listed in Table1.

**Table 2** shows the association between the SNPs and selenium status. *DMGDH* rs921943 was significantly associated with whole-blood selenium at 12 wk of gestation (*P*-adjusted = 0.03) and explained 2.0% of the variance, which was higher in individuals carrying the A allele. After adjustment for age, gestational age, and BMI, *DMGDH* rs921943 was also significantly associated with toenail selenium (*P* = 0.04), which explained 1.7% of the variance.

**TABLE 2:** Whole-blood selenium, toenail selenium, and GPx3 activity at baseline by genotype^1^

		Geometric mean (95% CI)^2^						
Matrix and SNP	Subjects, *n*	GG/CC	GA/CT	AA/TT	β ± SE	*R*^2^	*P*	*P*-adjusted^3^
Whole-blood selenium, μmol/L								
rs921943	227	1.29 (1.25,1.33)	1.36 (1.31, 1.40)	1.37 (1.27, 1.47)	0.016 ± 0.007	0.020	0.033	0.032
rs3877899	224	1.34 (1.30, 1.38)	1.30 (1.25, 1.34)	1.28 (1.17, 1.40)	−0.013 ± 0.008	0.012	0.101	0.148
rs7579	225	1.32 (1.28, 1.36)	1.32 (1.27, 1.37)	1.36 (1.27,1.46)	0.003 ± 0.007	7 × 10^−4^	0.696	0.511
rs1050450	225	1.34 (1.30, 1.38)	1.31 (1.27, 1.36)	1.31 (1.22, 1.40)	−0.006 ± 0.007	0.003	0.380	0.404
rs713041	225	1.32 (1.26, 1.37)	1.30 (1.27, 1.34)	1.38 (1.31, 1.45)	0.008 ± 0.007	0.005	0.282	0.260
Toenail selenium, μg/g								
rs921943	216	0.604 (0.588, 0.621)	0.624 (0.606, 0.643)	0.638 (0.601, 0.678)	0.012 ± 0.006	0.017	0.053	0.045
rs3877899	213	0.624 (0.609, 0.640)	0.602 (0.583, 0.622)	0.612 (0.568, 0.659)	−0.010 ± 0.007	0.010	0.151	0.236
rs7579	214	0.620 (0.605, 0.637)	0.606 (0.587, 0.625)	0.635 (0.596, 0.677)	−0.001 ± 0.006	5 × 10^−5^	0.918	0.910
rs1050450	214	0.604 (0.588, 0.622)	0.625 (0.607, 0.643)	0.629 (0.594, 0.665)	0.010 ± 0.006	0.013	0.099	0.086
rs713041	214	0.617 (0.595, 0.639)	0.617 (0.600, 0.633)	0.611 (0.586, 0.638)	−0.002 ± 0.006	4 × 10^−4^	0.775	0.796
GPx3 activity, U/L								
rs921943	225	72.5 (70.1, 75.0)	74.8 (72.2, 77.6)	73.5 (68.1, 79.4)	0.007 ± 0.007	0.003	0.387	0.342
rs3877899	222	74.0 (71.7, 76.3)	73.3 (70.4, 76.3)	71.0 (64.5, 78.1)	−0.006 ± 0.009	0.002	0.475	0.585
rs7579	223	73.1 (70.8, 75.6)	74.3 (71.5, 77.3)	73.1 (67.7, 78.8)	0.002 ± 0.008	3 × 10^−4^	0.790	0.667
rs1050450	223	74.3 (71.7, 77.0)	72.0 (69.5, 74.7)	76.6 (71.4, 82.2)	−0.0002 ± 0.008	5 × 10^−6^	0.974	0.914
rs713041	223	74.0 (70.7, 77.4)	73.3 (71.0, 75.8)	73.6 (69.7, 77.7)	−0.002 ± 0.008	2 × 10^−4^	0.845	0.929

1 All genetic analyses were carried out with the use of the PLINK v1.9 analysis toolset (20, 21), which performs a range of genetic analyses through a flexible regression framework. GPx3, plasma glutathione peroxidase; SNP, single nucleotide polymorphism.

2 All traits are presented on the natural scale for ease of interpretation.

3 Adjusted for age, gestational age, BMI at baseline, age at which education ceased (y), and social class (dichotomized).

Neither *SEPP1* nor glutathione peroxidase (*GPx*) polymorphisms were associated with whole-blood selenium or toenail selenium although there was a hint of an association between whole-blood selenium and *SEPP1* rs3877899 (*P* = 0.10) and of an association between *GPx1* rs1050450 and toenail selenium (*P* = 0.10). None of the polymorphisms was associated with GPx3 activity at baseline.

### Effect of genotype on longitudinal change in selenium status during pregnancy in the placebo group

We previously showed that whole-blood selenium decreased significantly (*P* < 0.0001) in the placebo group over the course of gestation (from 1.32 μmol/L at 12 wk of gestation to 1.16 μmol/L at 35 wk of gestation) (6). Neither *DMGDH* rs921943 nor either of the *GPx* genotypes had any effect on this change (**Table 3**). By contrast, the *SEPP1* rs3877899 genotype was significantly associated with the percentage change in whole-blood selenium from 12 to 35 wk of gestation, which explained 8% of the variance and decreased less in individuals carrying the minor A allele (Table 3). The association remained significant after adjustment for age, gestational age, and BMI at baseline (*P* = 0.005) and with allowance for multiple comparisons with the Bonferroni correction. Although the other *SEPP1* SNP rs7579 showed a tendency to affect the fall in the whole-blood selenium concentration over the course of gestation, the effect did not reach significance (*P*-adjusted = 0.088); the 2 *SEPP1* SNPs were not in significant linkage disequilibrium (*D*′ = 1, *r*^2^ = 0.124).

**TABLE 3:** Longitudinal changes in whole-blood selenium and GPx3 activity in the placebo group by genotype^1^

		Mean ± SD						
Variable change and SNP	Subjects, *n*	GG/CC	GA/CT	AA/TT	β ± SE	*R*^2^	*P*	*P*-adjusted^2^
Change in whole-blood selenium, %								
rs921943	109	−11.2 ± 8.4	−13.4 ± 10.4	−12.0 ± 10.2	−0.011 ± 0.014	0.006	0.423	0.504
rs3877899	108	−13.9 ± 10.0	−10.0 ± 7.2	0.3 ± 7.0	0.048 ± 0.016	0.080	0.003	0.005
rs7579	108	−11.0 ± 10.5	−13.2 ± 8.3	−16.5 ± 6.1	−0.025 ± 0.014	0.028	0.083	0.088
rs1050450	107	−11.6 ± 8.9	−12.6 ± 10.6	−13.7 ± 8.4	−0.011 ± 0.014	0.005	0.457	0.628
rs713041	108	−13.4 ± 8.6	−12.6 ± 9.9	−10.0 ± 9.6	0.016 ± 0.013	0.014	0.227	0.179
Change in GPx3 activity, %								
rs921943	107	0.4 ± 20.9	6.0 ± 30.1	3.9 ± 15.7	0.032 ± 0.037	0.007	0.393	0.402
rs3877899	106	1.2 ± 20.9	4.9 ± 30.4	18.8 ± 31.0	0.053 ± 0.044	0.014	0.236	0.128
rs7579	106	5.0 ± 26.2	1.7 ± 22.8	0.8 ± 30.5	−0.026 ± 0.039	0.004	0.505	0.484
rs1050450	105	−0.1 ± 22.1	8.1 ± 28.2	−2.1 ± 24.0	0.025 ± 0.038	0.004	0.509	0.658
rs713041	106	2.6 ± 29.7	4.2 ± 22.0	1.4 ± 26.8	−0.003 ± 0.036	9 × 10^−5^	0.923	0.876

1 All genetic analyses were carried out with the use of the PLINK v1.9 analysis toolset (20, 21), which performs a range of genetic analyses through a flexible regression framework. GPx3, plasma glutathione peroxidase; SNP, single nucleotide polymorphism.

2 Adjusted for age, gestational age, and BMI at baseline.

As we previously showed that there was no change in GPx3 activity from 12 to 35 wk of gestation in the placebo group (2), it was unsurprising that we saw no effect of genotype on change in GPx3 activity (Table 3).

### Effect of genotype on change in selenium status during pregnancy in the selenium-intervention group

We previously showed that whole-blood selenium increased significantly (*P* < 0.0001) in the selenium-treatment group over the course of gestation (from 1.31 μmol/L at 12 wk of gestation to 1.87 μmol/L at 35 wk of gestation) (6). However, neither *DMGDH* rs921943 nor any of the other polymorphisms were significantly associated with the percentage change in whole-blood selenium from 12 to 35 wk of gestation (**Table 4**).

**TABLE 4:** Changes in whole-blood selenium and GPx3 activity after selenium supplementation by genotype^1^

		Mean ± SD						
Variable change and SNP	Subjects, *n*	GG/CC	GA/CT	AA/TT	β ± SE	*R*^2^	*P*	*P*-adjusted^2^
Change in whole-blood selenium, %								
rs921943	104	42.3 ± 20.3	43.0 ± 20.1	43.1 ± 41.3	0.005 ± 0.033	2 × 10^−4^	0.890	0.871
rs3877899	102	42.1 ± 25.0	44.7 ± 19.9	39.9 ± 12.4	0.004 ± 0.035	1 × 10^−4^	0.914	0.879
rs7579	104	44.0 ± 23.5	43.7 ± 20.6	32.0 ± 21.9	−0.041 ± 0.032	0.015	0.209	0.230
rs1050450	104	40.2 ± 23.7	45.1 ± 21.9	43.1 ± 20.5	0.023 ± 0.031	0.006	0.454	0.455
rs713041	103	40.9 ± 16.1	42.0 ± 26.1	46.9 ± 20.5	0.028 ± 0.032	2 × 10^−4^	0.390	0.575
Change in GPx3 activity, %								
rs921943	104	9.5 ± 23.4	5.8 ± 25.3	−4.0 ± 18.8	−0.056 ± 0.036	0.023	0.121	0.144
rs3877899	102	2.0 ± 21.5	14.4 ± 26.9	13.7 ± 21.2	0.086 ± 0.036	0.053	0.020	0.010
rs7579	104	10.7 ± 25.0	1.9 ± 22.4	5.6 ± 20.8	−0.047 ± 0.034	0.018	0.180	0.187
rs1050450	104	9.5 ± 27.4	4.9 ± 18.0	5.3 ± 27.5	−0.027 ± 0.033	0.007	0.407	0.442
rs713041	103	3.6 ± 18.4	8.5 ± 28.3	7.7 ± 18.0	0.024 ± 0.035	0.005	0.487	0.622

1 All genetic analyses were carried out with the use of the PLINK v1.9 analysis toolset (20, 21),which performs a range of genetic analyses through a flexible regression framework. GPx3, plasma glutathione peroxidase; SNP, single nucleotide polymorphism.

2 Adjusted for age, gestational age and BMI at baseline.

In our previous study, we noted a significant increase in GPx3 activity from 12 to 35 wk of gestation in the selenium group (*P* = 0.01) (2). In the current study, we showed that that increase in GPx3 activity, which was expressed as a percentage change, was significantly associated with the *SEPP1* rs3877899 genotype (*P* = 0.01) and accounted for 5.3% of the variance and increased more in individuals with the minor A allele (Table 4). No other polymorphism was significantly associated with the percentage change in GPx3 activity.

## DISCUSSION

In a cohort of United Kingdom pregnant women, we showed significant associations between a polymorphism in *DMGDH* on chromosome 5q14 and baseline concentrations of whole-blood and toenail selenium, which validated our first hypothesis. Our finding replicated previous genome-wide association study results in populations of men and women that showed such associations either with blood (erythrocyte) selenium (15) or toenail -selenium (16) concentrations. We showed that concentrations of both whole-blood and toenail selenium were significantly associated with the *DMGDH* rs921943 genotype. The SNP explained 2.0% of the variation in whole-blood selenium and 1.7% of toenail selenium at 12–16 wk of gestation (the baseline in our study). Neither *SEPP1* nor *GPx* polymorphisms were significantly associated with whole-blood or toenail selenium at baseline.

The *DMGDH* rs921943 genotype did not affect the longitudinal change in selenium status in the placebo group or the response to supplementation in the selenium-supplemented group. However, both the change and response were significantly affected by SNP rs3877899 in the selenoprotein gene *SEPP1*, which validated our second and third hypotheses.

The *SEPP1* rs3877899 genotype affected the longitudinal fall in the whole-blood selenium concentration over the course of gestation, which explained a good proportion (8%) of the variance and decreased more in individuals carrying the G allele. Although this result warrants replication, it suggests that women carrying the minor A allele can maintain selenium status better than can women with the G allele during pregnancy. Furthermore, in contrast to the findings of an earlier study (12), the *SEPP1* rs3877899 genotype, but not the rs7879 genotype, significantly affected the response of GPx3 activity to selenium supplementation, explaining 5.3% of the variance and increasing more in carriers of the A allele. These observations suggest that women with the rs3877899 minor A allele can better maintain their circulating selenium concentration during pregnancy and are more responsive to selenium supplementation.

An alternative explanation is that women with the G allele preferentially synthesize SEPP1 (or other selenoproteins) rather than GPx3. SEPP1 has a special role in transferring selenium to the fetus in the latter half of gestation by means of a specific apolipoprotein E receptor 2 placental receptor (22). Hierarchical selenoprotein expression is known to occur during selenium deprivation and supplementation states (23–25). In our previous study in this same cohort, we concluded that supplemental selenium was probably being prioritized for the synthesis of SEPP1 rather than for GPx3; the concentration of SEPP1 at 35 wk of gestation was substantially higher in the selenium-treated group than in the placebo group, which was not the case for GPx3 activity (2). GPx3 activity remained at a very modest level compared with that in other pregnancy cohorts (2). If women with the GG genotype synthesize SEPP1 more readily than do those with GA or AA genotypes, we would have to assume that that SEPP1 was transferred to the fetus rather than remaining in the bloodstream; otherwise, the fall in whole-blood selenium that we observed would not have occurred.

The *DMGDH* rs921943 genotype only appears to affect baseline (steady state) selenium status. A considerable polygenic overlap has been shown between *DMGDH* rs921943 and other SNPs in the genes encoded by betaine homocysteine methyl transferase (*BHMT* and* BHMT2*) and cystathionine β-synthase (15, 16). All of these enzymes are involved in the metabolism of sulfur-containing amino acids, including methionine, by the methylation and demethylation reactions of the methionine cycle. The analogous selenium compounds, notably selenomethionine, which is the most common selenium species in food and selenium-yeast supplements (17), are probably similarly metabolized (15, 16). For instance, we know that selenomethionine is nonspecifically incorporated into proteins in place of methionine and that enzymes of the *trans*-sulphuration pathway also metabolize the seleno-analogs of homocysteine and cystathionine (26).

Selenium is excreted by metabolism to methylated products including trimethylselenonium ion [(CH_3_)_3_Se^+^] and the selenosugar 1-β-methylseleno-*N*-acetyl-d-galactosamine (8). The proportion of selenium excreted is dependent on the availability of methyl donors such as *S*-adenosylmethionine (27). One might consider that the greater the methylation capacity, the lower the proportion of selenium retained to contribute to a selenium status measurement. It is perhaps unsurprising that the variation at *DMGDH* (and potentially at *BHMT*,* BHMT2*, and cystathionine β-synthase) should affect seleno–amino acid metabolism and the concentration of selenium in blood or toenails in the steady state.

That the *SEPP1* rs3877899 genotype is associated with change in selenium status was not unexpected because this SNP is in the coding region for the SEPP1 protein, which is an important component of circulating selenium and a supplier of selenium for the synthesis of other selenoproteins including GPx3.

Our study had a number of limitations that need to be acknowledged. First, although we had measurements of the SEPP1 concentration at 35 wk of gestation, we had not measured it at baseline, and thus, we could not use it to test our hypotheses. Second, our only functional measure of selenium status was GPx3 activity; we had no other measures of selenoenzyme activity. Third, the pregnant population we studied was of relatively low selenium status, hence, findings may be different in populations of higher selenium status. Finally, because of the complexity and uniqueness of the study design, our results warrant replication in an independent study. The strengths of our study are that we were able to replicate, in pregnant women, the findings of earlier genome-wide association studies, and we saw a selenoprotein-genotype effect on longitudinal change in selenium status and the response to supplementation.

In conclusion, the *DMGDH* rs921943 genotype is significantly associated with selenium status in United Kingdom pregnant women. Women who carried the *SEPP1* rs3877899 minor A allele were better able to maintain their selenium status during pregnancy, and their GPx3 activity increased more when receiving selenium supplementation. Although these findings may suggest that women who carry the A allele are at an advantage when pregnant with regard to their selenium status, we have raised the possibility that the changes observed could be explained because these women are less able to prioritize the synthesis of SEPP1, which is an important source of selenium for the fetus. We plan to address the issue of pregnancy outcome in relation to genotype in a subsequent study.

## Footnotes

5 Abbreviations used: BHMT, betaine-homocysteine methyltransferase; DMGDH, dimethylglycine dehydrogenase; *GPx*, glutathione peroxidase; *GPx1*, cytosolic glutathione peroxidase; GPx3, plasma glutathione peroxidase; GPx4, phospholipid glutathione peroxidase; SEPP1, selenoprotein P; SNP, single nucleotide polymorphism; SPRINT, Selenium in Pregnancy Intervention.
